# UHRF1 predicts poor prognosis by triggering cell cycle in lung adenocarcinoma

**DOI:** 10.1111/jcmm.15438

**Published:** 2020-06-03

**Authors:** Zhenbo Tu, Xinzhou Deng, Shengqi Hou, Anlin Feng, Qiuping Zhang

**Affiliations:** ^1^ Department of Immunology School of Basic Medical Science Wuhan University Wuhan China; ^2^ Department of Clinical Oncology Taihe Hospital Hubei University of Medicine Shiyan China; ^3^ Human Oncology and Pathogenesis Program Memorial Sloan Kettering Cancer Center New York NY USA; ^4^ Department of Internal Medicine College of Medicine‐Phoenix University of Arizona Phoenix AZ USA

**Keywords:** cell cycle, lung adenocarcinoma, non‐small cell lung cancer, prognosis, ubiquitin‐like with plant homeodomain and ring finger domains 1

## Abstract

Accumulating evidence suggests that ubiquitin‐like with plant homeodomain and ring finger domains 1 (UHRF1) is overexpressed in non‐small cell lung cancer (NSCLC); however, the expression and function of UHRF1 in the subtype of NSCLC are still unclear. Here, we investigate the expression and prognosis traits of UHRF1 in large NSCLC cohorts and explore the molecular characters during UHRF1 up‐regulation. We find that UHRF1 is predominantly overexpressed in lung squamous cell carcinoma (SCC). Surprisingly, the up‐regulated UHRF1 is only associated with the overall survival of lung adenocarcinoma (ADC) and knockdown of UHRF1 dramatically attenuates ADC tumorigenesis. Mechanically, we identify a hub gene that includes a total of 55 UHRF1‐related genes, which are tightly associated with cell cycle pathway and yield to the poor clinical outcome in ADC patients. What's more, we observe knockdown of UHRF1 only affects ADC cells cycle and induces cell apoptosis. These results suggest that up‐regulated UHRF1 only contributes to lung ADC survival by triggering cell cycle pathway, and it may be a prognostic biomarker for lung ADC patients.

## 1 INTRODUCTION

Ubiquitin‐like with plant homeodomain and ring finger domains 1 (UHRF1) also known as ICBP90 in human and Np95 in mice, works by explicit binding hemi‐methylated CG sites through its SET‐ and RING‐associated domain to play a vital role in performing and maintaining DNA methylation through recruiting DNMT1 to hemi‐methylated DNA sites in S phase, which is dependent on ubiquitination of histone H3 at lysine 23.^1^, ^2^, ^3^, ^4^, ^5^ UHRF1 is involved in multiple diseases through the regulation of methylation,^6^, ^7^ especially in tumours where the dysregulation of UHRF1 is frequently observed.^8^, ^9^, ^10^ In lung cancer, UHRF1 was significantly up‐regulated in NSCLC compared with normal lung tissues^8^ and was expressed preferentially in non‐ADC.^11^ However, whether the ectopic expression of UHRF1 is associated with poor prognosis in SCC or ADC, and the related molecular mechanism are still unclear.

Presently, massive studies have shared their results of expression profiling on the commonly used platform, which provide opportunity for computational prediction of biomarkers, drugs and its potential mechanisms.^12^, ^13^, ^14^ By investigating the previously published gene expression microarray data, we set out to identify the expression profile and prognosis value of UHRF1 in NSCLC and its subtype, respectively, and further to elucidate the mechanism of how UHRF1 affects the subtype of NSCLC patients' prognosis.

## 2 MATERIALS AND METHODS

### 2.1 Cell culture and transfection

The lung adenocarcinoma cell line (H1975) and lung squamous cell line (SK‐MES‐1) were purchased from ATCC and cultured in DMEM (Gibco) with 10% foetal bovine serum (Invitrogen) at 37°C in CO_2_ Incubator. To generate stable UHRF1 knockdown cells, the siRNA sequences: Negative siRNA Negative Control (5′‐TTCTCCGAACGTGTCACGT‐3′), siRNA‐UHRF1‐1 (5′‐TGTGGACCATGGGAATTTTTTCACA‐3′), and siRNA‐UHRF1‐2 (5′‐TACACGGGTAGTGGTGGTCGAGATC‐3′) were transfected into H1975 and SK‐MES‐1 cells using Lipofectamine 3000 (Invitrogen).

### 2.2 Western blotting

Western blot analysis was performed using standard techniques as described before with the primary anti‐UHRF1 (Boster) and anti‐GAPDH (Proteintech).

### 2.3 Cell proliferation assay

The UHRF1‐siRNA transfected cells were plated in 96‐well plates at 3000/well and allowed to adhere overnight. CCK‐8 solution (10 µL) was added to each well of the plate and incubated for another 2 hours in indicated days. The absorbance was measured using a Multilabel Plate Reader (Monobind Inc) at 450 nm.

### 2.4 Wound healing assay

The UHRF1‐siRNA transfected cells were digested and proceed cell counting, cells were seeded in 6‐well plates and cultured with DMEM. A 10 μL white micropipette tip was used to create vertical wound in the cell monolayer. Images of the wound edges were captured at time 0 and 72 hours using a SONY ILCE‐A6000L/B camera.

### 2.5 Transwell assays

Cell migration was investigated with modified Boyden chamber (Costar). The UHRF1‐siRNA transfected cells were digested and proceed to cell counting. A total of 5 × 10^4^ cells in 100 μL were added to the upper wells, the lower compartments were added with 500 μL 10% FBS medium allowed cell to migrate for 12 hours at 37°C. Using cotton swabs to remove the cells that remained in the upper chamber. The membrane of the upper chamber was fixed with methanol for 20 minutes and stained with 0.1% crystal violet solution for 15 minutes. Then, washed the membrane with PBS for three times, and pictures of the migrated cells were taken for counting by using ImageJ (National Institutes of Health).

### 2.6 Cell apoptosis and cell cycle analysis

The UHRF1‐siRNA transfected cells were digested and proceed cell counting, then cells were double‐stained with Annexin V‐FITC and PI (Absin) after treatment with docetaxel (10 nM) 24 hours. For cell cycle analysis, transient UHRF1‐siRNA transfected cells were fixed with 70% ice‐cold ethanol for 2 hours and stained with PI containing RNase A solution (Absin). Data were acquired using a Beckman CytoFlex flow cytometer and analysed using CytExpert software (Beckman).

### 2.7 Meta‐analysis

We searched GEO database by the following keywords: (‘NSCLC’ OR ‘Non‐small cell lung carcinoma’ OR ‘Non‐small cell lung cancer’). The inclusion criteria are the both NSCLC tissues/healthy control or two main histological types (ADC and SCC) were included in each dataset which contained more than 100 human samples. We extracted all data into a standardized form which included following items: the name of first author, publication year, country, number of patients and the mRNA levels of UHRF1.

### 2.8 Survival analysis

We divided samples into two groups (UHRF1 high expression and low expression) in accordance with the mean value of expression of UHRF1 aiming to estimate survival by Kaplan‐Meier method. All figures were drawn by GraphPad Prism 6.

### 2.9 Bioinformatics analysis

The limma package (https://bioconductor.org/packages/release/bioc/html/limma.html) was used to obtain the differentially expressed genes (DEGs) between non‐survivors and survivors in TCGA ADC (Provisional), TCGA ADC (Nature), and TCGA SCC (Provisional), and these genes were served as NSCLC's survival‐related genes in this research. The GO analysis was performed on the DEGs. We also carried out KEGG analysis to find the obvious altered pathways (*P* < .05) during UHRF1 increase. Based on the interaction relationship from KEGG, we built pathway and gene regulatory networks. The PPI data were extracted from the STRING database (https://string‐db.org) which was based on the protein interactions and signalling pathways, and the network was built by Cytoscape 3.6.1 application.

### 2.10 Statistical analysis

Results were expressed as means ± SD and data were analysed using a two‐sided unpaired Student's *t* test. For all analyses, * and *** indicated *P* < .05 and *P* < .001, respectively.

## 3 RESULTS

### 3.1 UHRF1 is overexpressed in NSCLC tissues

**FIGURE 1:**
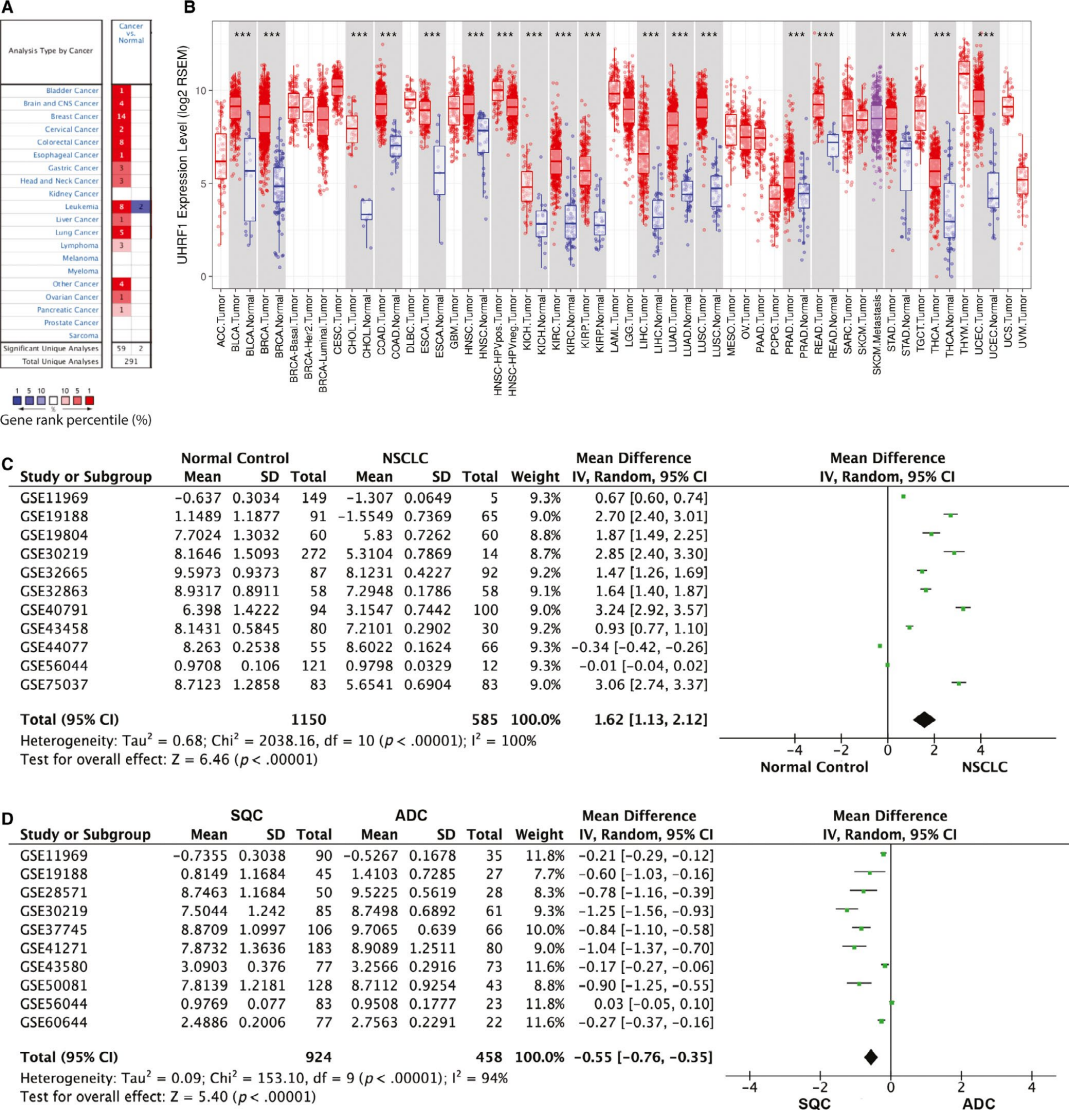
The expression of UHRF1 in different tissues. A, B, UHRF1 expression in multiple cancers and normal tissues from ONCOMINE data (A) and TIMER data (B); C, D, Forest plot for UHRF1 in NSCLC and normal lung tissues (C), ADC and SCC lung tissues (D)

We found UHRF1 was up‐regulated in multiple cancers compared with normal controls from ONCOMINE data (Figure 1A) and obtained the similar results from TIMER data derived from TCGA clinical patients (Figure 1B). Of interest, we focused on the role of UHRF1 in lung cancer; however, the UHRF1 expression in lung cancer was inconsistent from different groups. Thereby, we did a meta‐analysis based on the GEO data and total of 30 GEO datasets were assessed by titles or abstracts in GEO database up to August 2019. After checking the main texts carefully, 17 datasets with 2892 cases met with the standard of this research. We collected the following items from every study: first author, GEO accession number, year of publication, country and number of patients (Table S1). The mRNA levels of UHRF1 in NSCLC tumour tissues were significantly higher than that in healthy tissues, which was shown by meta‐analysis, and the pooled mean difference was 1.62 (11 datasets, 1735 patients, 95% CI 1.13–2.12, *Z* = 6.46, *P* < .00001, Figure 1C).

UHRF1 mRNA levels in ADC and SCC were also compared by meta‐analysis, and a statistical significance was observed (10 articles, pooled mean difference −0.55, 95% CI −0.76 to −0.35, *Z* = 5.4, *P* < .00001, Figure 1D). As shown in our analysis, the UHRF1 expression in SCC was significantly higher than that in ADC. These results indicate the UHRF1 expression is significantly up‐regulated in NSCLC, especially in SCC.

### 3.2 UHRF1 level is associated with NSCLC prognosis

**FIGURE 2:**
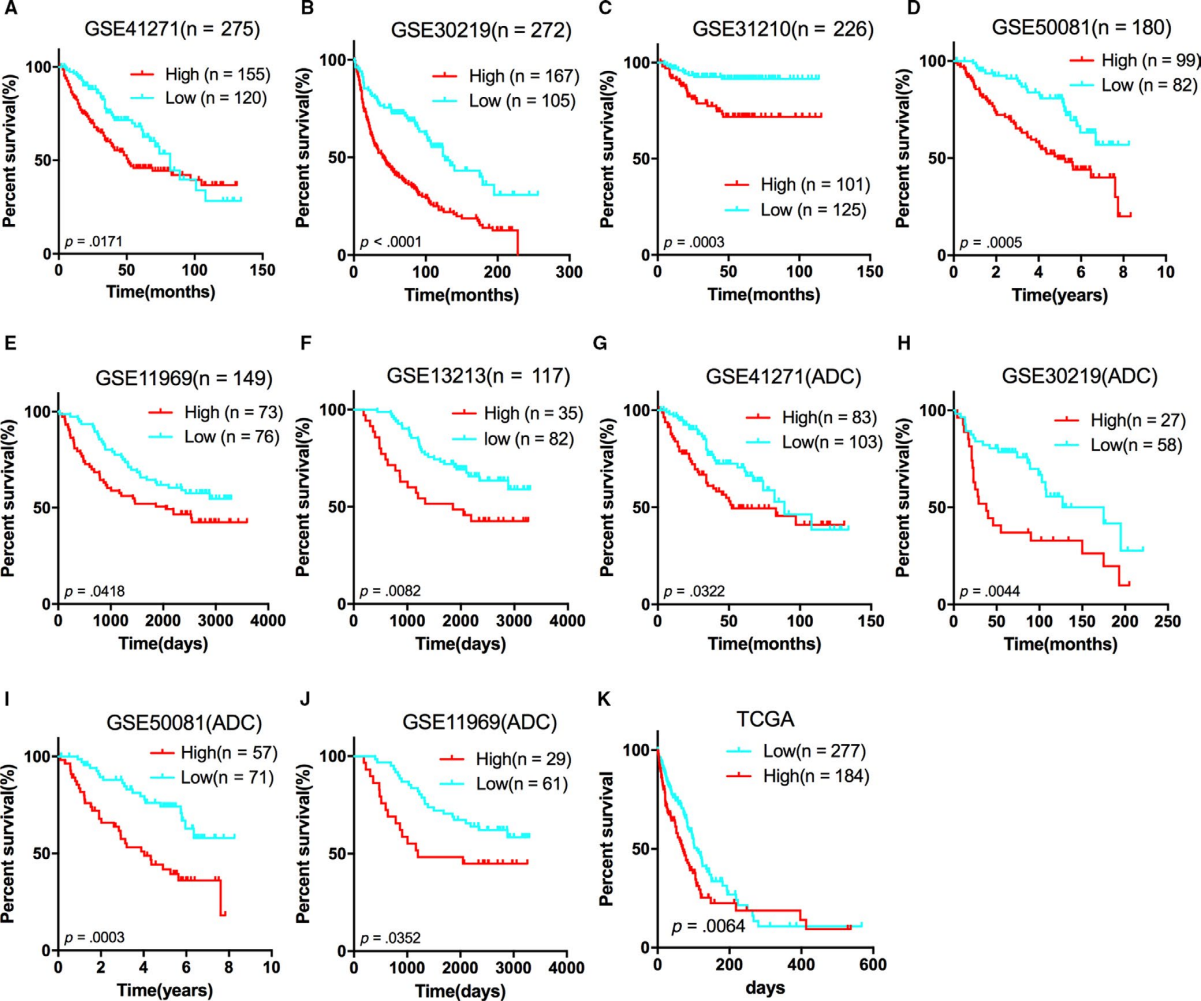
Kaplan‐Meier estimates of the survival of GEO patients according with UHRF1 expression. The Kaplan‐Meier plots were used to visualize the overall survival for the high UHRF1 expression vs low UHRF1 expression group of patients based on the mean value A, GSE41271; B, GSE30219; C, GSE31210; D, GSE50081; E, GSE11969; F, GSE13213; G, GSE41271; H, GSE30219; I, GSE50081; J, GSE11969; K, TCGA ADC Provisional. The tick marks on the Kaplan‐Meier curves represent the censored patients. The differences between the two curves were determined by the two‐sided log‐rank test

To identify whether UHRF1 expression affects the patient's survival, we divided NSCLC patients into high and low of UHRF1 expression group to perform survival analysis according to mean expression value of UHRF1. The results revealed that the patients with high expression of UHRF1 were associated with poor prognosis, indicated by GSE41271 (*P* = .0171), GSE30219 (*P* < .0001), GSE31210 (*P* = .0003), GSE50081 (*P* = .0005), GSE11969 (*P* = .0418) and GSE13213 (*P* = .0082) (Figure 2A‐F).

As a result of the observation that UHRF1 level in LCC and SCC was higher than that in ADC, so it would be interesting to know whether the UHRF1 expression in LCC and SCC played a more important role in prognosis. Therefore, we performed survival analysis about four GEO sets (GSE41271, GSE30219, GSE50081 and GSE11969) which included subclasses of NSCLC. But strikingly, there was no correlation of the UHRF1 expression in LCC and SCC with patients' survival in these four GEO sets (Figure S1). In turn, we found that high expression of UHRF1 was firmly associated with poor prognosis in ADC demonstrated by GSE41271 (*P* = .0322), GSE30219 (*P* = .0044), GSE50081 (*P* = .0003), GSE11969 (*P* = .0352) and TCGA ADC Provisional (*P* = .0064) (Figure 2G‐K).

### 3.3 Knockdown of UHRF1 attenuates ADC tumorigenesis

**FIGURE 3:**
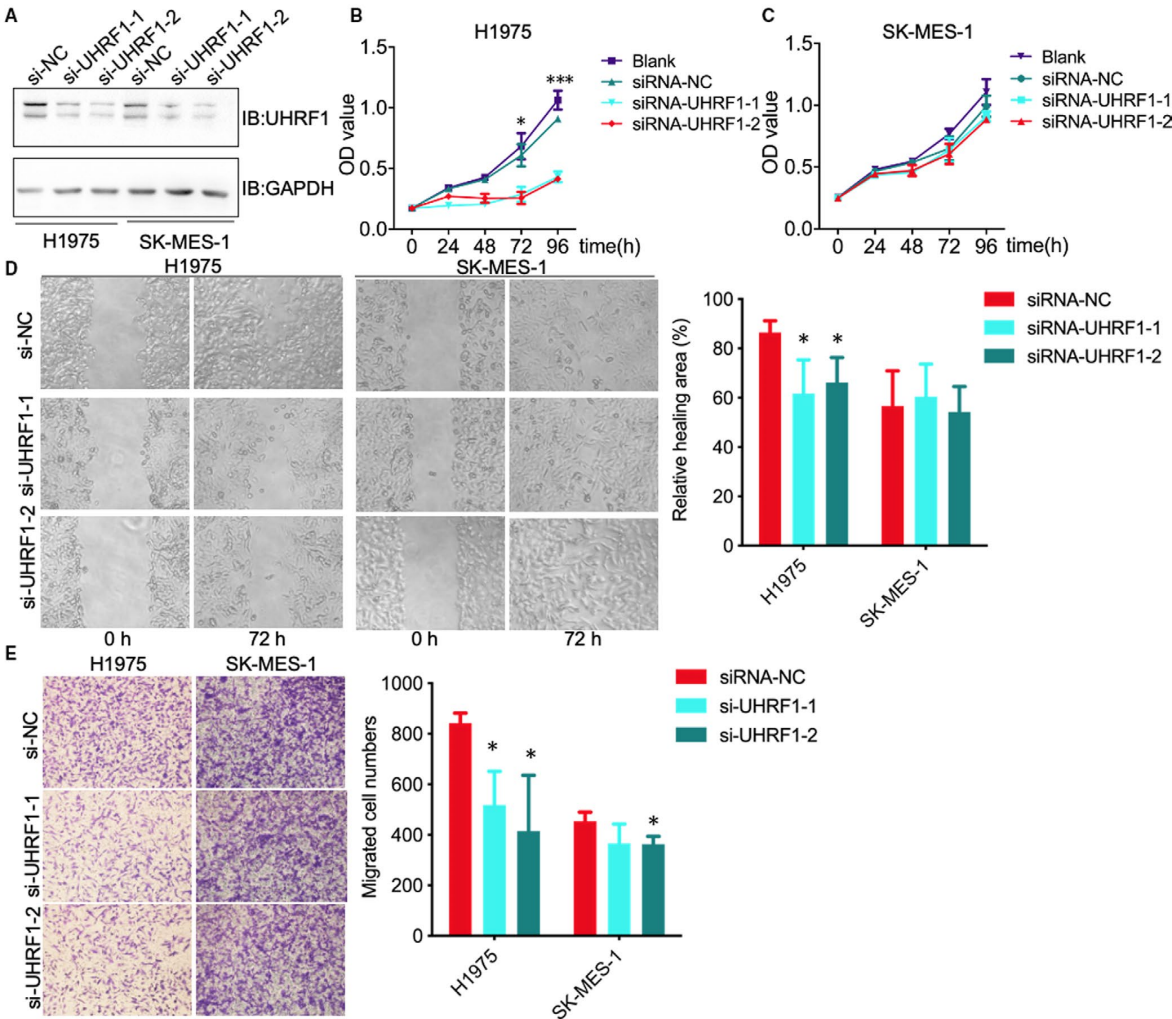
Knockdown of UHRF1 attenuates H1975 cells proliferation and migration. A, Representative blotting of UHRF1 and GAPDH in H1975 and SK‐MES‐1 cells after knockdown of UHRF1; B, C, Cell proliferation results of H1975 (B) and SK‐MES‐1 (C) cells after knockdown of UHRF1; D, Wound healing results of H1975 and SK‐MES‐1 cells after knockdown of UHRF1; E, Transwell analysis of H1975 and SK‐MES‐1 cell migration after knockdown of UHRF1, * and *** indicated P < .05 and P < .001, respectively

The conclusion that UHRF1 level only affects ADC prognosis was truly inconsistent with our expectation. As far as we know, poor prognosis is mainly caused by tumour metastasis and therapy resistance. Tumour metastasis yields approximately 90% cancer‐related death^15^ and includes multiple biologic steps, such as those of cell proliferation, migration, invasion, circulation, extravasation and colonization.^16^ Of interest, we investigated the biofunction of UHRF1 based on the knockdown of UHRF1 by using siRNA in ADC (H1975) and SCC (SK‐MES‐1) cells (Figure 3A). Indeed, knockdown of UHRF1 dramatically impeded H1975 cells growth (Figure 3B), but there was no any significant growth differences in SK‐MES‐1 cells (Figure 3C), which was consistent with the wound healing results (Figure 3D). What is more, both UHRF1 siRNA notably inhibited H1975 cells migration, especially the siRNA UHRF1‐2 inhibited around 50% of cell migration, but only the siRNA UHRF1‐2 decreased around 20% of cell migration in SK‐MES‐1 (Figure 3E). These results suggest knockdown of UHRF1 can dramatically impede ADC tumorigenesis when compared with SCC.

### 3.4 UHRF1 triggers cell cycle to yield poor prognosis in ADC

**FIGURE 4:**
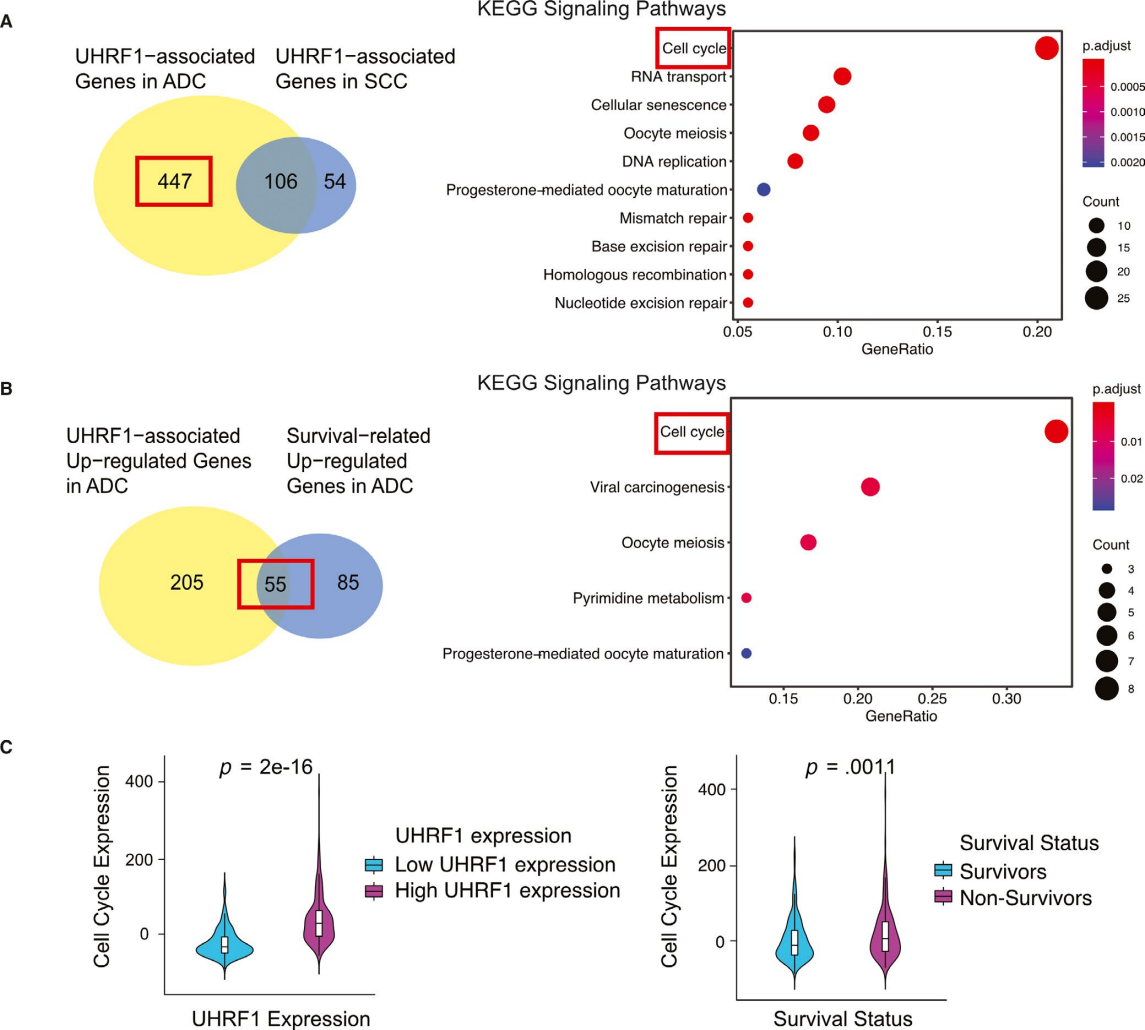
The bioinformatics analysis results of ADC patients in TCGA datasets. A, The Venn plot of UHRF1‐related genes from ADC and SCC, and KEGG pathways enriched among UHRF1‐associated genes in ADC; B, The Venn plot of UHRF1 up‐regulated genes and survival‐associated up‐regulated genes, and KEGG pathways enriched among intersection between UHRF1‐associated genes in ADC and ADC survival‐related genes; C, Cell cycle gene expression

So far, the results of survival analysis from GEO and TCGA indicated that UHRF1 mostly affects the survival and progression of ADC. However, the associated key pathways and genes in ADC are not so clear. Thus, we explored the signalling pathways and interacted proteins with UHRF1 based on bioinformatics methods in ADC (TCGA Provisional [discovery cohort], TCGA Nature [validation cohort]) and SCC (TCGA Provisional datasets). In contrast to the low level of UHRF1 group, there were 553 UHRF1‐associated genes in ADC and 160 UHRF1‐associated genes in SCC (Figure 4A). More UHRF1‐related genes were found in ADC which might indicate that UHRF1 had more transcriptome impact on the ADC. In general, 447 genes (263 up‐regulated and 184 down‐regulated genes in UHRF1 high expression samples) were exclusively changed in ADC discovery cohort.

We used the DAVID Functional Annotation Bioinformatics Microarray Analysis to identify enriched KEGG pathways among the 447 genes and found cell cycle signalling pathways, RNA transport, and cellular senescence et al were enriched among 263 up‐regulated genes (Figure 4A). No pathways were found as being enriched among down‐regulated genes. So, we chose 263 up‐regulated genes for further research.

To confirm the genes which have the same regulation direction in high UHRF1 and non‐survivor patients, we intersected the 263 genes with 140 survival‐related up‐regulated genes in ADC and detected 55 protein‐coding genes in the intersection which were both related with UHRF1 and ADC survival. These 55 protein‐coding genes might not only be a potential gene biomarker for ADC patients but also a key factor of differentiating the SCC and ADC survival. Results showed that DEGs were significantly enriched cell cycle from GO and KEGG pathway (Figure 4B). The expression of cell cycle pathway in non‐survivors was significantly higher than that in survivors, and the result was detected between high and low UHRF1 expression patients (Figure 4C).

**FIGURE 5:**
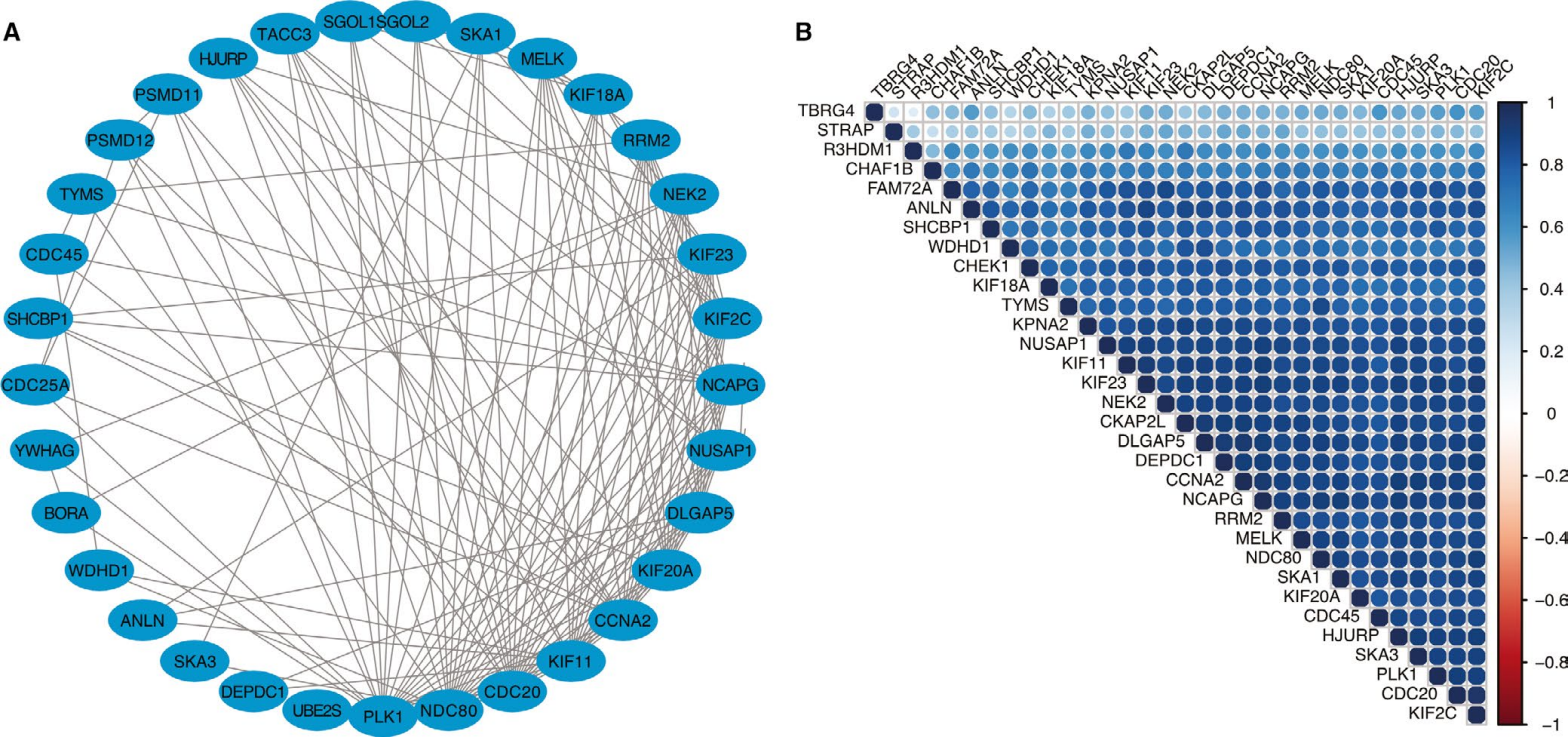
UHRF1‐related genes' interaction network (A) and gene co‐expression matrix (B)

After the discovery of the significantly varied pathways, protein and protein interaction (PPI) and correlation analysis were used to identify the interaction between these 55 proteins. We showed that one strong network among 55 proteins which had a stronger enriched network than that in random proteins, and these genes were especially associated with cell cycle pathway (Figure 5A). The correlation matrix owing correlation coefficients between sets of genes were derived from ADC discovery cohort, and it revealed that most proteins from this network had strong positive correlations with each other (Figure 5B). So, these genes established by UHRF1‐associated genes, especially cell cycle‐related genes have a strong interwork interaction and it could be used as a multi‐gene biomarker for predicting the survival of NSCLC patients and hub genes for differentiating the survival status difference of high UHRF1 in ADC and SCC.

### 3.5 The prediction score of hub genes predicts ADC clinical outcome

**FIGURE 6:**
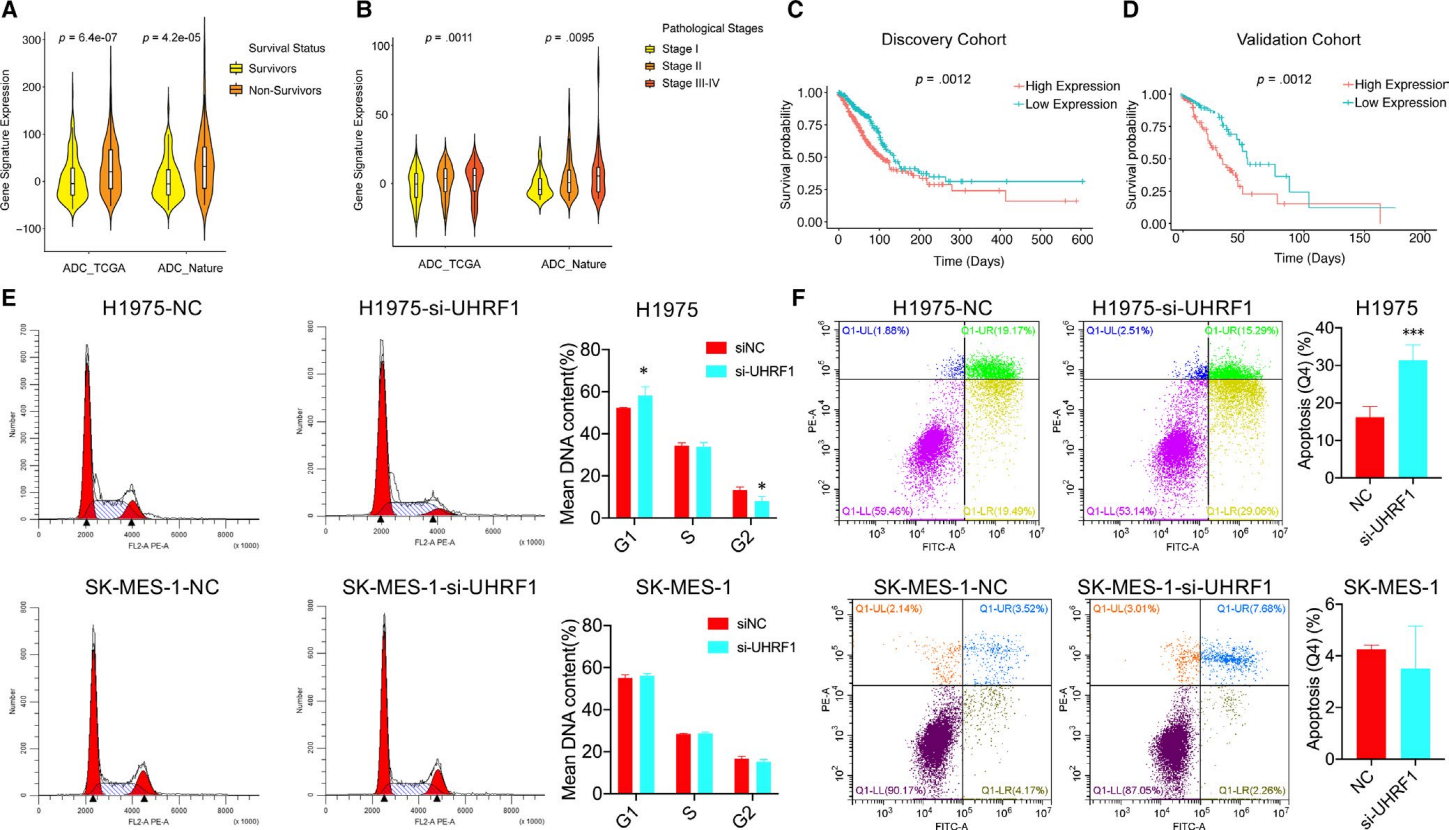
UHRF1‐related genes in ADC could predict the survival and pathological status of ADC patients. A, The gene signature expression in survivors and non‐survivors; B, The gene signature expression in different pathological stages; C, Kaplan‐Meier plot in ADC discovery and validation cohort; D, Kaplan‐Meier plot in ADC validation cohort; E, H1975 and S‐MES‐1 cells were transfected with siRNA‐UHRF1‐1 and FACS analysis was performed to detect the cell cycle distribution; F, The H1975 and S‐MES‐1 cells were transfected with siRNA‐UHRF1‐1 and then treatment with docetaxel, and FACS analysis was performed to detect the cell apoptosis, * and *** indicated P < .05 and P < .001, respectively

We utilized a survival prediction score based on the expression of the 55 genes to verify whether the 55 genes have the sufficient power to predict the death risk of ADC and SCC patients. To make the prediction feasible with one unique ‘survival prognosis score’, a scoring system representing a linear combination of the 55‐gene expression with a weight value was constructed to allocate each patient with a score to measure the possibility of risk (Weight values for each gene are based on the direction of differential expression: 1 for the up‐regulated and −1 for the down‐regulated genes in non‐survivors. The risk score of each patient was obtained from sum of multiplication of normalized expression value of the gene by its corresponding weight value). A higher risk score represented a worse clinical outcome. Our results focused on both the ADC discovery/validation cohort and SCC TCGA datasets. Interestingly, the scores from non‐survivors were significantly higher than those of survivors both in the ADC discovery and validation cohort (Figure 6A), there were not any significant differences in SCC cohort (Figure S2). Same results were obtained in ADC (discovery *P* = .0011, validation *P* = .0095) and SCC cohort according to the pathological stage (Figure 6B). The Kaplan‐Meier plot confirmed the survival results in both discovery and validation cohorts (Figure 6C,D). Notably, we observed the G1 phase was increased while G2 phase was decreased in H1975 after knockdown of UHRF1, but there was not any significant differences in SCC SK‐MES‐1 cells (Figure 6E). In the meantime, we observed significant apoptosis of ADC H1975 cells after treatment with docetaxel in UHRF1‐down‐regulation group; however, there were no effects in SK‐MES‐1 cells (Figure 6F). These results indicate that UHRF1 is likely to trigger cell cycle. Based on all these results, the 55 genes detected from UHRF1 expression had the statistical power to predict clinical outcome in ADC and thus elucidated why the high UHRF1 expression in ADC and SCC had diverse clinical outcome.

## 4 DISCUSSION

With the popularity of whole‐genome sequencing in recent years, more and more sequencing data have been uploaded to public data platforms. Based on these data, we performed meta‐analysis to identify UHRF1 expression in NSCLC and its subclasses and, found the UHRF1 positive rate in SCC and LCC were higher than that in ADC. To our surprise, UHRF1 was specifically associated with poor prognosis of ADC. To find the underlying molecular mechanisms to explain this phenomenon, we defined 55 genes derived from UHRF1 network as our hub genes which can predict both survival rate and pathological stage of lung ADC patients.

One of our most striking findings was the relative molecular difference caused different clinical outcome in lung ADC and SCC patients. We used this result as our clue to detect all the UHRF1‐related proteins which can affect the survival, especially in ADC. Surprisingly, these proteins also possessed the power to predict the survival and pathological stage in ADC. Recently, several studies investigated that histology subtypes determine different treatments. Confirming all the genetic differences between ADC and SCC will have the potential impact for treatment. Some NSCLC new treatments like EGFR tyrosine kinase inhibitors and ALK inhibitors have better benefits to patients with ADC than SCC.^17^, ^18^ Thus, what we found in this study not only provided a potential UHRF1‐targeted therapy in lung ADC in future, but also revealed the differences in molecular pathways among lung ADC and SCC.

According to previous studies about how UHRF1 affects the progression of cancer, we made two conclusions on the molecular mechanisms: Firstly, UHRF1 plays an important role in transferring DNA methylation status from mother cells to daughter cells. It can recognize hemi‐methylated DNA which appears in freshly synthesized daughter DNA strands by the SRA domain during DNA replication, then recruits DNMT1 to ensure faithful maintenance of DNA‐methylation patterns in daughter cells.^6^ That is, the overexpression of UHRF1 can induce the DNA hypo‐methylation of UHRF1 hub genes in ADC. DNA hypo‐methylation can lead to oncogenesis through several molecular mechanisms like unstable chromosomal, aberrant gene expression including oncogenes and tumour suppressor genes. Secondly, UHFR1 depletion in cancer cells causes cell cycle arrest in G2/M‐phase,^19^ while Jenkins et al^20^ also show reduction of UHRF1 decreases the growth rates in several tumour cell lines. In view of these results, we postulate that UHRF1 is essential for proliferation in human cancer cell.

In conclusion, our study indicates that UHRF1 is overexpressed in NSCLC, especially in SCC, but the up‐regulated UHRF1 only contributes to ADC patients' survival by activating cell cycle pathway. These findings could benefit the understanding of the effect of UHRF1 on NSCLC and reveal the potential targets for NSCLC subclasses individual prognosis and treatment.

## CONFLICT OF INTEREST

The authors declare no conflicts of interests.

## AUTHOR CONTRIBUTION

**Zhenbo Tu:** Project administration (equal); Writing‐original draft (lead). **Xinzhou Deng:** Project administration (equal). **Shengqi Hou:** Writing‐review & editing (supporting). **Anlin Feng:** Conceptualization (equal); Supervision (equal); Writing‐review & editing (lead). **Qiuping Zhang:** Conceptualization (equal); Supervision (equal).

## Supporting information

Supplementary Material

## Data Availability

The data that support the findings of this study are available from the corresponding author upon reasonable request.
