# Supplementary material for: UHRF1 predicts poor prognosis by triggering cell cycle in lung adenocarcinoma
Source: J Cell Mol Med. 2020 Jun 3;24(14):8069–77. doi: 10.1111/jcmm.15438 (PMC7348181; doi:10.1111/jcmm.15438)

**Supplementary Table 1**

Table 1 Main characteristics of included studies in the meta-analysis.

| **Author** | **GEO Accession Number** | **Year** | **Country** | **Patients Number** |
| --- | --- | --- | --- | --- |
| Botling J | GSE37745 | 2013 | Sweden | 196 |
| Der SD | GSE50081 | 2014 | Canada | 181 |
| Girard L | GSE75037 | 2016 | USA | 166 |
| Hou J | GSE19188 | 2010 | Netherlands | 156 |
| Kabbout M | GSE43458 | 2013 | USA | 110 |
| Kadara H | GSE44077 | 2014 | USA | 226 |
| Karlsson A | GSE56044 | 2014 | Sweden | 136 |
| Karlsson A | GSE60644 | 2014 | Sweden | 117 |
| Kim IJ | GSE32665 | 2013 | USA | 179 |
| Lu TP | GSE19804 | 2010 | Taiwan | 120 |
| Matsuyama Y | GSE11969 | 2011 | Japan | 163 |
| Micke P | GSE28571 | 2011 | Sweden | 100 |
| Rousseaux S | GSE30219 | 2013 | France | 307 |
| Sato M | GSE41271 | 2013 | USA | 275 |
| Selamat SA | GSE32863 | 2012 | USA | 116 |
| Tarca AL | GSE43580 | 2013 | Switzerland | 150 |
| Zhang Y | GSE40791 | 2012 | USA | 194 |

**Supplementary Figure legend**

Supplementary Figure 1. UHRF1 overexpression correlated with poor survival in patients with LCC and SQC. A: GSE41271(SQC); B: GSE30219(LCC); C: GSE30219(SQC); D: GSE50081(LCC); E: GSE50081(SQC); F: GSE11969(LCC); G: GSE11969(SQC).

Supplementary Figure 2. The bioinformatics analysis results of SQC patients in TCGA datasets. A. The level of UHRF1 in death and survivors group; B. The level of UHRF1 in different pathological stage; C. The survival probability of different level of UHRF1.

Supplementary Figure 1

Supplementary Figure 2


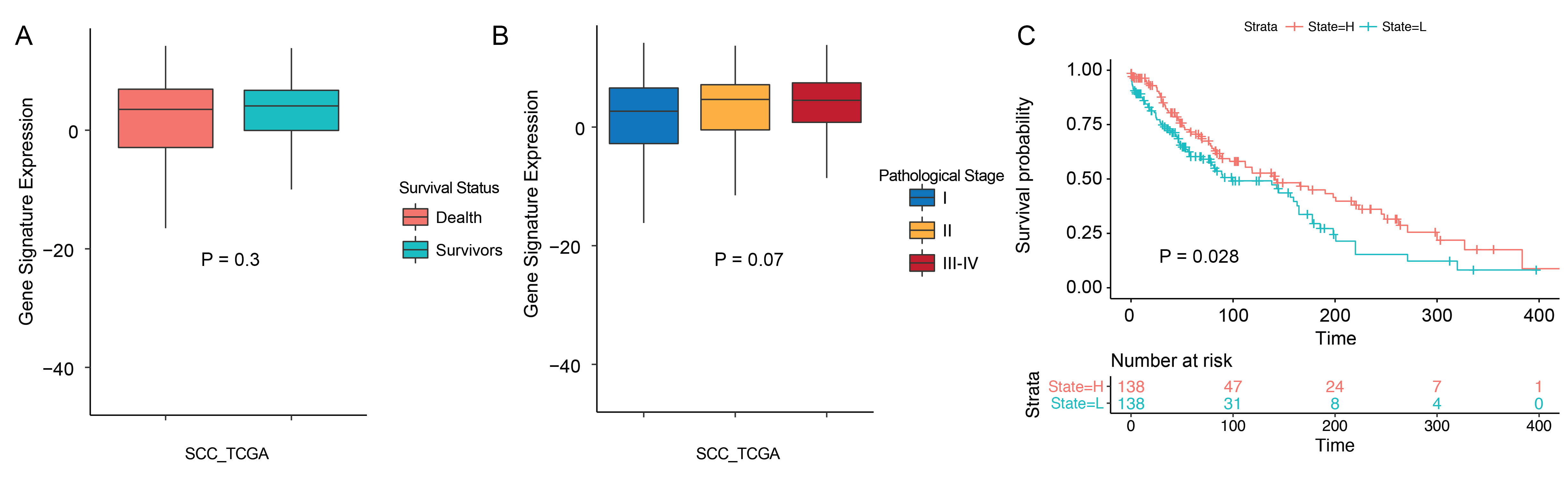

Supplement: Supplementary file 1 — Supplementary Material [file JCMM-24-8069-s001.docx]
